# Genetic frequencies related to severe or profound sensorineural hearing loss in Inner Mongolia Autonomous Region

**DOI:** 10.1590/1678-4685-GMB-2015-0218

**Published:** 2016-10-10

**Authors:** Yongzhi Liu, Liying Ao, Haitao Ding, Dongli Zhang

**Affiliations:** 1 Department of Otolaryngology, Inner Mongolia People's Hospital, Hohhot, China.; 2 Department of Laboratory Medicine, Inner Mongolia People's Hospital, Hohhot, China.; 3 Department of Otolaryngology, the 4th Affiliated Hospital of Inner Mongolia Medical University, Hohhot, China.

**Keywords:** Sensorineural hearing loss, GJB2, SLC26A4, GJB3, mitochondrial DNA, Inner Mongolia

## Abstract

The aim was to study the frequencies of common deafness-related mutations and their contribution to hearing loss in different regions of Inner Mongolia. A total of 738 deaf children were recruited from five different ethnic groups of Inner Mongolia, including Han Chinese (n=486), Mongolian (n=216), Manchurian (n=24), Hui (n=6) and Daur (n=6). Nine common mutations in four genes (*GJB2, SLC26A4, GJB3* and mitochondrial *MT-RNR1* gene) were detected by allele-specific PCR and universal array. At least one mutated allele was detected in 282 patients. Pathogenic mutations were detected in 168 patients: 114 were homozygotes and 54 were compound heterozygotes. The 114 patients were carriers of only one mutated allele. The frequency of *GJB2* variants in Han Chinese (21.0%) was higher than that in Mongolians (16.7%), but not significantly different. On the other hand, the frequency of *SLC26A4* variants in Han Chinese (14.8%) was lower than that in Mongolians (19.4%), but also not significantly different. The frequency of patients with pathogenic mutations was different in Ulanqab (21.4%), Xilingol (40.0%), Chifeng (40.0%), Hulunbeier (30.0%), Hohhot (26.3%), and in Baotou (0%). In conclusion, the frequency of mutated alleles in deafness-related genes did not differ between Han Chinese and Mongolians. However, differences in the distribution of common deafness-related mutations were found among the investigated areas of Inner Mongolia.

## Introduction

Hearing loss is the most common sensory impairment that affects normal communication and life quality of the patients. About 70% of hearing loss is non-syndromic, and the remaining 30% is considered syndromic, accompanied by additional clinical abnormalities. Approximately 1 in 1,000 children is affected by prelingual hearing loss. The sensorineural hearing loss (SNHL) has been reported to occur in 2 of all newborns, and the ratio increases to 2.7 in 4 years-old children, and to 3.5 at puberty (Qing *et al*., 2015).

Various genetic and environmental causes are involved in the etiology of hearing loss (Hone and Smith, 2001, Mahboubi *et al*., 2012), which has increased in some places (Morton, 2002, Khabori and Patton, 2008, Sajjad *et al*., 2008). Genetic causes alone explain 50–70% of all cases (Petit *et al*., 2001, Smith *et al*., 2005). Genetic defects may cause susceptibility to environmental factors that contribute to late-onset deafness. Among a number of deafness-related genes (the Hereditary Hearing Loss Homepage, http://hereditaryhearingloss.org/), mutations in *GJB2*, *SLC26A4*, *GJB3*, and mitochondrial *12S rRNA* gene (*MT-RNR1*) are found to be more frequent. Mutations in *GJB2* and *SLC26A4* have been reported to cause autosomal recessive nonsyndromic hearing loss, while the inheritance pattern of *GJB3* mutations is not quite clear (Nishio and Usami, 2015). Mutations in these genes are estimated to explain more than 40% of non-syndromic hearing loss cases (Wu *et al*., 2011, Qing *et al*., 2015).

Several common mutations have been identified in the four hearing loss-related genes. Ethnic differences exist in the genetic pathogenesis of deafness. In Caucasian, Asian, Ashkenazi Jew, and African populations, the most common mutations in the *GJB2* gene are c.35delG, c.235delC, c.167delT, and c.427C > T, respectively. The second most common gene related to SNHL is *SLC26A4*. Mutations in this gene are considered major causes for enlarged vestibular aqueduct (EVA). Similar to *GJB2*, the frequency of *SLC26A4* mutations also varies in different populations. The c.919-2A > G is a highly prevalent mutation in East Asian subjects but is rare in European lineages (Dai *et al*., 2009).

Microarray chip, a high-throughput and high-efficiency technology, has been widely used in gene mutation detection. Here in this study, we used a microarray chip developed by CapitalBio Corporation (Beijing, China) to detect deafness-related mutations. This microarray chip aims to detect frequent mutations that are related to hereditary hearing loss in Chinese population, as seen in large-scale epidemiological studies done across China (Wang *et al*., 2007, Dai *et al*., 2008, Li *et al*., 2008, Chen *et al*., 2011). It enables simultaneous detection of nine common mutations in four genes of hereditary hearing loss, including c.235delC, c.35delG, c.176del16, c.299-300delAT in *GJB2*, c.IVS7-2A > G, c.2168A > G in *SLC26A4*, c.538C > T in *GJB3*, and m.1555A > G, m.1494C > T in *mtDNA 12S rRNA* Gene (*MT-RNR1*).

China is a country with multiple ethnicities. A recent research has shown that frequencies of mutations in *MT-RNR1* (1555A > G or 1494C > T) in a group of multiethnic minorities was almost six times higher than that in the Han group of southwestern China. The authors studied mainly ethnic groups more prevalent in southwestern China, such as Miao, Tujia, Dong and Zhuang (Qing *et al*., 2015). Mongolians account for an important part of the Inner Mongolia population and make up for about 17.11% according to China's Sixth Population Census in 2010 (http://www.chinadaily.com.cn/china/2010census/). Han Chinese account for 79.54%, while the remaining minorities account for 3.36%. However, little is known about the frequency of mutations in the four deafness-related genes in Inner Mongolia, especially considering the two main ethnic groups (Han Chinese and Mongolian). In this study, we analyze nine common gene mutations (*GJB2*, *SLC26A4*, *GJB3* and *MT-RNR1*) in 738 children with severe or profound hearing loss in Inner Mongolia. Ethnic and regional differences were also studied.

## Materials and Methods

### Subjects recruitment and clinical evaluation

**Figure 1:**
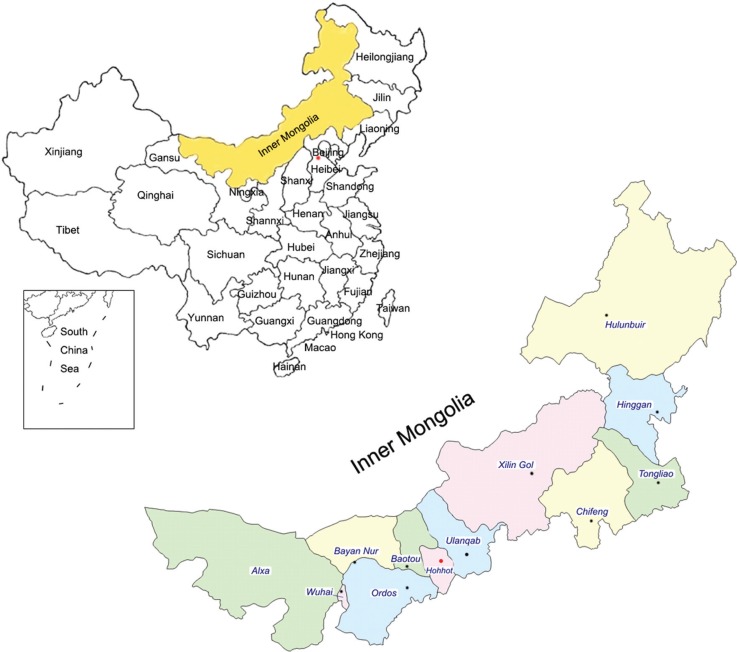
Map of China (upper portion) and Inner Mongolia (lower portion), showing the area from where patients were recruited.

From February 2011 to January 2013, 738 children with severe or profound hearing loss (456 male; 61.8%) were recruited for this study. All patients presented with congenital sensorineural deafness. Subjects from 12 main cities/leagues of Inner Mongolia Autonomous Region (Table 1) were selected in the Inner Mongolia People's Hospital, where they were treated with cochlear implants. The geographical areas from where subjects were recruited are shown in Figure 1. The 738 children with hearing impairment were all born in Inner Mongolia and belonged to five different ethnic groups, including Han Chinese (n=486), Mongolian (n=216), Manchurian (n=24), Hui (n=6) and Daur (n=6). The mean age of the participants was 4.24 ± 3.03 years, ranging from 12 months to 16 years. Clinical and general information was recorded including family history and past medical history. The study was performed under the approval of the Ethics Committee of Inner Mongolia People's Hospital. Signed informed consent was obtained from the legal guardians of the children.

**Table 1:** Characteristics of study participants.

Characteristic	Number of patients
**Gender**	
Male	456
Female	282
**Ethnics**	
Han Chinese	486
Mongolian	216
Manchurian	24
Hui	6
Daur	6
**Cities/leagues**	
Hohhot	114
Baotou	72
Bayannur	34
Ulanqab	84
Xilingol	30
Chifeng	150
Tongliao	108
Hinggan	60
Hulunbeier	60
Ordos	7
Alxa	12
Wuhai	7

All participants underwent audiological assessment, such as pure tone audiometry (PTA), auditory brainstem response (ABR), and multiple auditory steady-state evoked responses (ASSR). High-resolution thin-section computed tomography (HRTSCT) and magnetic resonance imaging (MRI) were also carried out. Exclusion criteria were as follows: (1) conductive deafness; (2) hereditary syndromic deafness; (3) acute or chronic otitis media; (4) deafness with a clear cause like late Meniere's disease, acoustic neuroma, meningitis, ototoxic drugs and trauma; and (4) children with systemic diseases.

### Genetic analysis

Genetic analysis was conducted for all patients using genomic DNA from peripheral blood samples. The genetic screening included nine common mutations in four common non-syndromic hearing loss related genes, including *GJB2* (NG_008358.1), *SLC26A4* (NG_008489.1), *GJB3* (NG_008309.1; NM_024009.2) and *MT-RNR1* (NC_012920.1). The mutations were detected by allele-specific polymerase chain reaction (PCR) and universal array (BioMixer^TM^, CapitalBio Corporation, Beijing, China) for simultaneously screening the nine mutations leading to hearing impairment (*GJB2*: c.235delC, c.35delG, c.176del16, c.299-300delAT; *SLC26A4*: c.919-2A > G, c.2168A > G; *GJB3*: c.538C > T; *MT-RNR1*: m.1555A > G, m.1494C > T). The multiplex allele-specific PCR was performed as described previously (Qu *et al*., 2012) and the results of microarrays were scanned and analyzed by a LuxScan^TM^ 10K/B Microarray Scanner (CapitalBio). The results were also validated by Sanger sequencing for wild and mutant types.

### Statistical analysis

Data was analyzed using SPSS software (version 17.0). Statistical significance was evaluated using an χ^2^ test to compare the differences among the ethnic groups and cities/leagues. P < 0.05 was considered to be statistically significant.

## Results

### Mutation frequencies of common deafness genes

At least one mutated allele was detected in 282 of the 738 deaf children, including 162 cases with *GJB2* mutations, 114 cases with *SLC26A4* mutations, and 6 with *GJB3* mutations. Mutations of *MT-RNR1* (m.1555A > G, m.1494C > T) were not detected in the sample. The frequencies of mutant alleles are shown in Table 2. Each allele was counted individually for compound heterozygotes. Thus, patients with two different mutated alleles were counted twice. Among all variants detected, the most frequent mutated alleles were *GJB2* c.235delC (41.8%) and *SLC26A4* c.919-2A > Ge (32.7%) (Table 2).

**Table 2:** Frequencies of the nine common deafness-associated alleles.

Gene/allele	Mode of inheritance	Homozygous	Heterzygous^a^	Total	Frequency in all mutated alleles (%)
*GJB2*	AR				
c.235delC		54	84	138	41.8
c.176-191del16		0	24	24	7.27
c.299-300delAT		0	42	42	12.7
c.35delG		0	0	0	0
*SLC26A4*	AR				
c.IVS7-2A > G		60	48	108	32.7
c.2168A > G		0	12	12	3.64
*MT-RNR1*	Maternal inheritance				
m.1555A > G		0	0		0
m.1494C > T		0	0		0
*GJB3*					
c.538C > T		0	6	6	1.82
Total		114	216	330	100

AR: autosomal recessive; AD: autosomal dominant.

a Heterozygous in different genes were counted separately.

### Frequencies of mutations in Han Chinese and Mongolians

The frequency of mutated alleles in *GJB2* in Han Chinese (21.0%, 102/486) was higher compared to that in Mongolians (16.7%, 36/216), but without significant difference (P=0.217). On the other hand, the frequency of mutated alleles in *SLC26A4* was lower in Han Chinese (14.8%, 72/486) than in Mongolians (19.4%, 42/216), also not significantly different. Our results suggest that the frequencies of the most common deafness-related mutations and genes are similar in Han and Mongolian groups (Table 3).

**Table 3:** Frequency of the mutated alleles in the four deafness-related genes screened in Han Chinese and Mongolian ethic groups.

Mutated Genes		Han Chinese	Mongolian	P-value
*GJB2*	Positive	102	36	0.217
	Negative	384	180	
*SLC26A4*	Positive	72	42	0.149
	Negative	414	174	

### Mutations in different cities/leagues

As shown in Table 4, 168 patients showed pathogenic mutations, of which 114 had homozygous mutations and 54 were compound heterozygotes. The 114 patients were called "carriers" since they presented only one mutated allele. The mutation frequencies were higher in four cities/leagues including Ulanqab, Xilingol, Chifeng and Hulunbeier. In Hohhot, 30 patients (26.3%) were diagnosed as homozygotes or compound heterozygotes. However, in Baotou, a city located less than 200 kilometers away from Hohhot, no pathogenic mutation was detected. The frequencies were significantly different between the two main cities in Inner Mongolia, suggesting a difference in the geographical distribution of common deafness-related mutations.

**Table 4:** Patients with different deafness-related mutations from different cities/leagues in Inner Mongolia.

Cities/leagues	Patients No. (n)	Pathogenic Mutation		Carrier
		Homozygous	Compound heterozygous	Heterozygous
Hohhot	114	12 (10.5%)	18 (15.8%)	12 (10.5%)
Baotou	72	0	0	6 (8.3%)
Bayannur	34	0	6 (17.6%)	0
Ulanqab	84	12 (14.3%)	6 (7.1%)	30 (35.7%)
Xilingol	30	6 (20%)	6 (20%)	6 (20%)
Chifeng	150	42 (28%)	18 (12%)	30 (20%)
Tongliao	108	18 (16.7%)	0	6 (0.06%)
Hinggan	60	6 (10%)	0	12 (20%)
Hulunbeier	60	18 (30%)	0	12 (20%)

## Discussion

Mutations in various hearing loss-related genes have been studied worldwide, and several genes have been identified as commonly related to hearing impairment, such as *GJB2*, *SLC26A4*, *GJB3* and *MT-RNR1* (mtDNA 12S rRNA gene). In the present study, mutation analysis was performed in 738 children with severe to profound SNHL in Inner Mongolia. Similar results have been reported by many studies. After screening for mutations of three prominent deafness-related genes (*GJB2*, *SLC26A4* and *mtDNA 12S rRNA*) in 235 patients with hearing loss in the Yunnan province of China, 35.74% of patients showed genetic involvement, and 17.45%, 9.79%, and 8.51% of inherited hearing impairment were caused by *GJB2*, *SLC26A4*, and *mtDNA 1555A > G* mutations, respectively (Xin *et al*,. 2013). Mutations of the *GJB2*, *SLC26A4*, and *mtDNA* have also been screened in 1164 children with severe or profound SNHL in southwestern China. Approximately 28% (331/1164) of patients were found to carry mutations, of which 17.27%, 7.04%, and 4.12% were *GJB2*, *SLC26A4*, and *mtDNA*, respectively (Qing *et al*., 2015). *GJB2* c.235delC and *SLC26A4* c.919-2A > G have been widely known to be the most common mutations in Chinese population (Qu *et al*., 2012, Zhang *et al*., 2013). Similarly, we also found that the frequencies of *GJB2* c.235delC and *SLC26A4* c.919-2A > G among all detected mutated alleles were the highest.

The pathogenicity of *GJB3* mutations has not been defined, and it is still unclear whether these mutations are related to autosomal dominant or recessive nonsyndromic hearing loss, especially in the case of mutation c.538C > T. The mutation in *GJB3* (c.538C > T) has been first identified to be associated with autosomal dominant hearing impairment in two Chinese families (Xia *et al*., 1998). However, in a carrier screening for normal hearing female populations of childbearing age in South China, 9 subjects (in 7,263 women, 0.12%) were found carrying *GJB3* c.538C > T in a heterozygous state, suggesting that it might act as an autosomal recessive mutation (Yin *et al*., 2013). Moreover, the variant c.538C > T has been recategorized as "benign" lately, based on allele frequency (Shearer *et al*., 2014). Considering the uncertainty of the pathogenicity of this variant, patients with heterozygous *GJB3* c.538C > T were counted as "carriers" in our study. Shearer *et al*. (2014) proposed a threshold of 0.0005 for considering a variant as pathogenic in an autosomal dominant fashion, in the general population. However, the allelic frequency was 0.004 (6/1476 alleles) in our study, suggesting a possible association with deafness. Further studies are needed to elucidate the pathogenicity of *GJB3* variant c.538C > T.

Previous reports have indicated that the prevalence of *GJB2* mutations varies among different ethnic groups. The most common *GJB2* mutation in Caucasians (c.35delG) has also been the most frequent mutation in patients of Uyghur ethnicity in Xinjiang province, where central and western Asian cultures converge on this northwestern border of China (Chen *et al*., 2011). The c.35delG has not been detected in our patients that were from five different ethnic groups in Inner Mongolia (Han, Mongolian, Manchurian, Hui and Daur). However, the c.235delC accounted for 41.8% of all *GJB2* mutations detected in our study.

Inner Mongolia is located on the northern border of China, and is populated by a variety of ethnicities, but mainly by Han and Mongolian ethnicities. We found no significant difference in the frequency of *GJB2* and *SLC26A4* mutations between Han and Mongolian ethnicities. Similar results have been reported in southwestern China study, where no significant difference in frequencies of *GJB2* and *SLC26A4* mutations among China minorities and Han ethnicity populations were detected (Qing *et al*., 2015).

Although the frequencies of common deafness-related mutations are similar in many reports, local differences still exist. In our study, we found significant differences among different cities/leagues in Inner Mongolia. At least one mutated allele was detected in more than 50% of patients in four cities/leagues including Ulanqab, Xilingol, Chifeng and Hulunbeier. Interestingly, a significant difference in the frequency of deafness-related mutations was found between Hohhot and Baotou. This suggests a strong difference in mutation distribution. A possible explanation may be traced back to the beginning of the Ming and Qing dynasties, when a large population migration movement called "Zou Xikou" started (Yin and Bai, 2013). The migration from Shanxi province to Inner Mongolia lasted more than 300 years, and promoted the formation of Baotou. This migration might have contributed to the difference in frequencies of deafness-related mutations between Hohhot and Baotou. Further studies with more participants involved are needed to verify the results and elucidate possible causes.

The reported frequencies of common mutations in *GJB2*, *SLC26A4*, *GJB3* and *MT-RNR1* in children with SNHL in Inner Mongolia, present a conclusive molecular diagnosis of hearing loss. The low mutation frequency in patients from Baotou suggests that other mutations in other deafness-associated genes may explain hearing loss in this area.
